# EVALUATING A MICROLEARNING INTERVENTION ABOUT NUTRITIONAL CARE FOR OLDER PEOPLE TARGETED AT NURSES

**DOI:** 10.1093/geroni/igad104.1485

**Published:** 2023-12-21

**Authors:** Debbie ten Cate, Jeroen Dikken, Roelof Ettema, Lisette Schoonhoven, Marieke Schuurmans

**Affiliations:** Utrecht University of Applied Sciences, Utrecht, Utrecht, Netherlands; The Hague University of Applied Sciences, The Hague, Zuid-Holland, Netherlands; Utrecht University of Applied Sciences, Utrecht, Utrecht, Netherlands; University Medical Center Utrecht, Utrecht University, Utrecht, Utrecht, Netherlands; Dutch Healthcare Authority, Utrecht, Utrecht, Netherlands

## Abstract

Nurses play a pivotal role in the care of older people with malnutrition or who are at risk. However, research shows that they lack the motivation to give adequate nutritional care. To change this motivation, an educational intervention targeted at nurses would be desirable. To optimize learning efficacy, we developed a microlearning intervention and conducted a first evaluation. The aim of this study was to evaluate the microlearning intervention on reaction, learning and behavioral change among nurses. The evaluation was assessed with focus groups, a questionnaire, participant observations and patient records. The data was both qualitatively and quantitatively analyzed. Two focus groups were held with a total of seven nurses. They mostly mentioned positive experiences, stated that their knowledge about nutritional care increased and that their behavior in daily practice changed. Of the 306 participants, who received the intervention, 94 filled in the questionnaire of which 66% was satisfied with the microlearning intervention and over 69% agreed that they learned something new. Five nurses were observed while giving care to a total of twelve older care recipients. No clearly detectable changes in behavior during care moments or in reporting in patient records were observed. Nurses had a mainly positive experience with the microlearning intervention. They confirmed learning and changing behavior in daily practice. However, from observations this behavioral change was not visible. The intervention may be potentially useful in education for (future) nursing professionals. With the microlearning intervention, nurses may be better equipped to provide nutritional care to older people.

